# “Mirror, Mirror, Am I Beautiful?” Mechanisms of Self-Image Cognition and Behavioral Responses Among Chinese Youth in the Context of Digital Beauty Filter Use: A Mixed-Methods Study Using Grounded Theory and fsQCA

**DOI:** 10.3390/bs16071082

**Published:** 2026-07-01

**Authors:** Chao Zhang, Yinze Hao, Jing Li

**Affiliations:** 1 School of Journalism and Communication, Henan University, No. 379, North Section of Mingli Road, Zhengzhou 450046, China; steven@henu.edu.cn (C.Z.); haoyinze@henu.edu.cn (Y.H.); 2 Journalism and Information Communication School, Huazhong University of Science and Technology, No. 1037 Luoyu Road, Hongshan District, Wuhan 430074, China

**Keywords:** Chinese youth, digital beauty filters, self-image cognition, behavioral responses, grounded theory, fsQCA

## Abstract

As digital beauty filters have become widespread among young people, their links with self-image cognition and behavioral responses have attracted growing attention. However, existing studies largely focus on Western samples or linear approaches, leaving Chinese youth underexplored in their cultural context. Using mixed methods combining grounded theory and fsQCA, this study examines the mechanisms shaping self-image cognition and behavioral responses among Chinese youth in the context of digital beauty filter use. Semi-structured interviews and three-stage coding identified four core categories: Beauty Filter Use Habits, Beauty Filter Use Motivations, Beauty Filter Use Preferences, and Psychological Responses to Beauty Filter Use. Building on this, fsQCA identified five configurational pathways. The psychological-response–motivation and psychological-response–preference core coexistence configurations were linked to high Self-Image Cognition; the three non-high Self-Image Cognition pathways formed two patterns: dual absence of psychological responses and motivations, and motivational-core absence with coexisting habits and preferences. Different self-image cognition outcomes may relate to adaptive behaviors, such as moderate retouching and naturalized self-presentation, or risk-related behaviors, such as avoidance of original images and overdependence on beauty filters. This study offers a new perspective on youth authentic self-construction and technological adaptation in the digital visual era, with implications for media literacy education, platform design, and mental health intervention.

## 1. Introduction

With the rapid development of artificial intelligence and visual computing, the production, editing, and circulation of digital images are being profoundly reshaped (H. Chen et al., 2025). Digital beauty filters (hereafter, “beauty filters”), as a key product of this evolution, have shifted from specialized photo-editing tools to infrastructures embedded in everyday imaging practices (D. Yang et al., 2021). From selfies and short videos to AI-based retouching, beauty filters have entered everyday life in accessible and instantaneous ways. Through facial adjustment, skin-tone enhancement, and related functions, individuals can directly reprocess their own images, turning appearance from something recorded into something editable and optimizable. While enhancing visual expression, however, beauty filters have also subtly changed how individuals perceive, present, and evaluate themselves. As retouched images increasingly enter everyday interactions, individuals’ understanding of the relationship between their real and virtual selves may also shift. When “retouching before posting” becomes habitual, technological processing moves beyond image beautification and enters the domain of self-cognition, becoming linked to judgments about appearance, self-worth, and identity. This cognitive shift has given beauty filters more complex social consequences. On the one hand, continuously optimized images may widen the gap between real and ideal appearance, trapping individuals in cognitive dilemmas of appearance anxiety (McLean et al., 2015), in some contexts spilling over into offline behaviors such as cosmetic surgery intentions (J. Chen et al., 2019). On the other hand, beauty filters are increasingly intertwined with appearance-based consumption and platform commercialization, turning personal image into a consumable resource (Zeng & Qian, 2025) and generating new traffic channels and consumption scenarios in beauty consumption, fitness content production, and livestream commerce (Y. L. Wang et al., 2023). Beauty filters therefore create a tension-filled context across cognitive pressure, behavioral spillover, and consumer practice. Among various technology user groups, youth are among the most active users of digital technologies, as their learning, social interaction, and everyday practices are closely coupled with online platforms (Steinsbekk et al., 2024). With the popularization of social media and platformized communication, beauty filters have extended from selfie retouching to social sharing, short-video posting, and even job-related self-presentation, becoming deeply embedded in young people’s social, everyday, and work practices (Chua & Chang, 2016). For many young people, beauty filters are no longer merely optional add-ons but have become an important part of everyday image production and self-presentation. Meanwhile, youth is a critical stage for the development of self-identity and body image (Arnett, 2000). In a context shaped by mianzi, or “face,” culture and social evaluation pressure, beauty filter use increasingly extends beyond aesthetic choice and becomes a practice of responding to real-world expectations (L. Zhou & Zhang, 2017). It is therefore evident that beauty filters have become deeply involved in the formation of self-image cognition among Chinese youth. However, how young people understand the relationship between technologically processed images and the real self, and how this cognitive process is further associated with their behavioral responses, remains to be further revealed.

## 2. Literature Review

### 2.1. Research on How Media Technologies Influence Youth Behavior

In the era of digital intelligence, media technologies have become deeply embedded in young people’s everyday lives, continuously reshaping their behavioral logic across multiple dimensions (Piccerillo & Digennaro, 2025). This influence is first reflected in the construction of social relationships, as media technologies function not only as channels of communication but also as active mediators of how young people establish, maintain, and negotiate relationships (Steinsbekk et al., 2024). Especially during transitional stages such as school progression and enrollment, young people often use social media to build connections, position their identities, and integrate into communities, making media platforms important sites of everyday interaction and emotional attachment (Thomas et al., 2017). Such relational practices are further intertwined with young people’s self-presentation behaviors. Self-presentation on social media shapes young people’s behavioral strategies in expression, feedback, and comparison, prompting them to adjust how they post content, respond to interactions, and display their images (C.-C. Yang et al., 2017). As ability-based and social comparisons accumulate on platforms, young people may repeatedly recalibrate their self-presentation and interaction choices through continuous comparison. Research on Chinese youth aged 18–44 further shows that social media use intensity is associated with real-world pressures such as employment anxiety through mechanisms including upward social comparison, online social support, and self-esteem (Jin et al., 2024). This suggests that platform-based comparison has extended to young people’s judgments of real-world competition and their own perceived competence. The behavioral role of media technologies therefore no longer remains limited to modes of communication but further enters the concrete processes through which young people manage relationships and express themselves in everyday life (C.-C. Yang et al., 2018). In a deeply mediatized context, young people’s everyday practices have also changed markedly. Some young people actively adopt quantified media technologies, incorporating self-monitoring and self-discipline into technology-supported routines through purchase, embedding, and integration, thereby developing lifestyles centered on efficiency and order (Y. Yang & Jiang, 2025). However, technological embeddedness may also generate reverse adaptation. Faced with constant connectivity, information overload, and a loss of control over daily rhythms, some young people turn to digital minimalism, seeking to regain control through digital decluttering and boundary resetting (Xu & Zhu, 2023). In addition, research on Chinese working youth further shows that digital device use significantly affects young people’s bedtime and sleep quality (Zhao & Wu, 2022), indicating that the influence of media technologies has extended from online interaction to their sleep–wake rhythms, health status, and everyday behavioral regulation. Media technologies have further altered the motivational structure of youth behavior formation. Social media influencers have become an important force shaping young people’s consumer attitudes, lifestyle choices, and value preferences; their influence extends beyond explicit recommendations and gradually permeates everyday decision-making through sustained attention and emotional involvement (Lajnef, 2023). Meanwhile, this influence does not always produce one-dimensional behavioral outcomes. Fans’ deep engagement with influencers may also trigger more attachment-oriented problematic interactions through parasocial relationships and a sense of belonging (Farivar et al., 2022). More broadly, the frequent intervention of media technologies may also generate risks of behavioral dysregulation. Problematic social media use and social media addiction may affect not only young people’s interpersonal relationships but also their daily behavioral regulation and developmental trajectories (Lin et al., 2023) and may further undermine academic performance, social interaction, and interpersonal ties; platforms such as Facebook and Instagram are common sites in which such problematic behaviors emerge (Pellegrino et al., 2022). Yet young people are not merely passive recipients of technology. They also actively use platforms to obtain social and emotional support and adjust their behavior across different media contexts, demonstrating considerable agency in media use (Gibson & Trnka, 2020).

Existing studies have partly examined the multiple relationships between media technologies and youth behavioral patterns. However, most have focused on relatively macro- or meso-level technological forms and media environments, such as artificial intelligence, social media platforms, or problematic use, while paying limited attention to micro-level technologies that are embodied and deeply embedded in everyday expression. This limitation becomes especially evident when technological influence extends to how young people understand, perceive, and present themselves; in such cases, a general behavioral perspective is no longer sufficient.

### 2.2. Research on the Sociocultural Impacts of Digital Beauty Filters

Digital beauty filters generally refer to real-time retouching technologies embedded in social media or image applications that use digital overlays, parameter adjustment, and algorithmic recognition to instantly enhance users’ physical image features and align their presented images with beautified ideals (Dijkslag et al., 2024). With technological advancement, beauty filters have moved beyond mere image retouching and have gradually become involved in the shaping of contemporary sociocultural life. One of the earliest shifts concerns how social actors understand “beauty”. Although digital filters can simulate diverse visual styles, their algorithms parameterize aesthetics, turning style from individualized expression into something that can be reproduced at scale (Qin, 2021). As this parametric logic becomes embedded in everyday shooting, sharing, and circulation, aesthetic standards are homogenized through technological mediation. The narrow ideal of being thin, fair-skinned, and youthful is promoted by appearance-rating algorithms, automated through facial beautification algorithms, and repeatedly reinforced as an existing bias (C. F. Chen & Shi, 2022). This bias also unfolds in culturally differentiated ways. Chinese women report significantly greater perceived body-image discrepancies than American women, revealing cultural differences in beauty filter use motivations and body imaginaries (D. Yang et al., 2021). Meanwhile, racial biases common in Digital beauty filters on social media, through skin lightening and facial-feature modification, continue to encode Eurocentric beauty standards into everyday visual circulation (Riccio et al., 2024). Changes in aesthetic logic further extend to body perceptions and social interaction. Digital filters allow individuals to directly reprocess their own images, shifting appearance from something recorded to something editable and optimizable (Zeng & Qian, 2025). As the body increasingly enters visual experience as something modifiable, decomposable, and reconstructable, the natural and organic body is gradually replaced by a social body shaped by technological logic (Liu, 2019). This transformation further extends into labor and interactional contexts. In livestreaming, for example, beauty filters have become basic labor tools for streamers, regulating intimate distance and disciplining bodily presentation among platforms, guilds, and audiences (Y. L. Wang et al., 2023). As beauty filters become embedded in everyday communication, they redefine self-presentation and interpersonal interaction. While beautified images support impression management and relational negotiation, the technology itself shifts from a tool to a mediating force in social interaction (Bhandari & Bimo, 2022). Cross-cultural research shows that users of video face filters often employ beauty filters for front-stage presentation in public settings, transforming filter use into an important resource for digital self-presentation through audience management, appearance optimization, and the pursuit of social recognition (Herring et al., 2025). This influence is especially pronounced among young people, who are actively engaged in self-construction, peer interaction, and social display, making the expressive pleasure, interactive connection, and appearance feedback enabled by filters more easily integrated into everyday self-presentation (Szambolics et al., 2023). Technological intervention may even subtly rewrite the relationship between young people’s “I” and “me.” By turning the real body into a digital image that can be viewed, compared, and revised, filters make young people more likely to look back at themselves from an objectified perspective, blurring the boundary between real and virtual bodies (Dijkslag et al., 2024). Such effects become more pronounced in platform environments where the body and appearance are highly visualized. A study of Chinese women (aged 18–44) on Xiaohongshu further found that appearance-oriented content is associated with behavioral risks such as body dissatisfaction, compulsive exercise, and disordered eating through psychological mechanisms including appearance comparison and state self-esteem (X. Wang et al., 2026). However, this process does not unfold in a one-dimensional manner. Different motivations for use show distinct associations with individual well-being and self-acceptance, suggesting that beauty filters operate through more complex sociocultural mechanisms (Javornik et al., 2022).

Overall, existing research has partly revealed the cultural influence of beauty filters on aesthetic standards, body perceptions, and social interaction, and has begun to note their particular relevance to youth. However, most studies have focused on general sociocultural consequences, leaving insufficient systematic analysis of how beauty filters intervene in individuals’ self-image cognition through concrete use practices and further relate to their behavioral responses. In the Chinese cultural context in particular, further inquiry is needed into how young people form understandings and evaluations of their appearance, overall image, and self-worth across real, retouched, and ideal images, and how these processes relate to concrete behavioral practices.

### 2.3. Problem Statement

Beauty filters have become deeply embedded in young people’s everyday practices, yet existing research has rarely offered a systematic account of how their concrete use processes intervene in self-image cognition among Chinese youth and become further associated with related behavioral responses. Accordingly, this study focuses on Chinese youth and examines the dynamic mechanisms of self-image cognition and behavioral responses in the context of digital beauty filter use. To this end, this study combines grounded theory with fuzzy-set qualitative comparative analysis (fsQCA), conducting the research at two levels: identifying key antecedent conditions and analyzing configurational pathways. The following research questions are proposed:

RQ1: What key conditions are associated with self-image cognition among Chinese youth in the context of digital beauty filter use?

RQ2: How do these conditions form different configurational pathways associated with self-image cognition among Chinese youth?

RQ3: How are different configurational pathways further associated with young people’s behavioral responses?

## 3. Research Design

This study adopts a sequential exploratory mixed-methods design to examine the mechanisms of self-image cognition and related behavioral responses among Chinese youth in the context of digital beauty filter use, following a progressive pathway of “qualitative exploration—condition transformation—configurational analysis—interpretive return to the materials.” In the first stage, grounded theory was used for qualitative exploration. Semi-structured interviews were conducted to collect experiences of beauty filter use, and open coding, axial coding, and selective coding were applied to identify key categories in beauty filter use that were associated with self-image cognition, thereby providing the conditional basis for the subsequent fsQCA analysis.

In the second stage, fsQCA was used to analyze configurational pathways. Based on the key categories generated from the qualitative analysis, this stage transformed them into antecedent conditions and set self-image cognition as the direct outcome variable, examining the associations between different configurations and self-image cognition outcomes. The configurational pathways identified through fsQCA did not replace qualitative interpretation; rather, they were returned to the interview materials to interpret the behavioral responses potentially linked to different states of self-image cognition. Thus, the two stages were not independent or parallel, but formed a continuous integration in which qualitative materials generated conditions, quantitative configurations presented pathways, and interview materials explained behavioral responses.

## 4. Grounded Theory Analysis

### 4.1. Interview Sample and Data Collection

Data for this study were collected between November 2025 and January 2026. Regarding the definition of the sample age range, iResearch Consulting’s China Beauty Imaging App Users’ Marketing Value Insight Reports released in 2020, 2022, and 2024 show that the proportion of users aged over 36 among core users has increased year by year, indicating a certain age extension in the user base of beauty imaging apps (iResearch Consulting, 2020, 2022, 2024). Meanwhile, with the rapid development of Chinese society, changes such as the disruption of career continuity by the gig economy, cultural feedback from digital natives, and the diversification of gender identities have meant that youth in China is no longer understood merely as a narrow biological age category, but is being redefined through multiple factors, including biological attributes, social roles, and cultural symbols (Xie, 2026). Across domestic and international scholarship, studies in multiple fields have also begun to extend the age definition of Chinese youth to 44. For example, some scholars examining the relationship between social media use and subjective well-being among Chinese youth have adopted a Chinese youth sample with an upper age limit of 44 (Y. Zhou & Wang, 2023). Other scholars, in studying the relationship between digital activity and sleep problems among Chinese working youth, have also defined their research subjects as Chinese youth with an upper age limit of 44 (Zhao & Wu, 2022). In addition, the World Health Organization, drawing on relevant research in fields such as psychology and medicine, defines individuals aged 18–44 as youth (Han, 2022). Based on these considerations, this study ultimately included Chinese youth aged 18–44 who use beauty filters. For interview sampling, this study combined purposive and snowball sampling. Participants were eligible if they were born between 1981 and 2007, had experience using digital beauty filters or related tools, and were able to discuss their use experiences and image evaluations. Recruitment information was posted through online communities, social media platforms, and personal networks, and interviewees were invited with consideration of gender, age, occupation, education, and beauty filter use experience. Participants who had completed the interview were also asked to recommend eligible individuals, thereby expanding the sample sources. Semi-structured interviews were conducted using an interview guide developed from existing literature and revised after pilot interviews with five young researchers who had experience using beauty filters. A total of 45 interviews were completed. By the 40th interview, the core categories had become stable; five additional interviews were then conducted, and data collection ended after no new concepts emerged. All interviews were conducted with informed consent, including 22 offline interviews and 23 online video interviews. Each interview lasted 45–70 min, with an average duration of approximately 60 min. All interviews were audio-recorded and transcribed, yielding approximately 380,000 Chinese characters of textual data. Because this study concerns subjective experiences related to beauty filter use, self-presentation, and image evaluation, the analysis remained attentive to participants’ specific accounts and use contexts. The researchers had backgrounds in media behavior and digital technology use research, but had no direct teaching, supervisory, or evaluative relationship with the participants. Category development was refined through repeated reading of the transcripts, comparison with original statements, and team discussions. To protect privacy, all participants were anonymized: male participants were coded with M and female participants with F, as shown in Table 1.

### 4.2. Open Coding

Open coding is a foundational step in grounded theory for extracting key information from raw data and forming concepts, with the aim of identifying and refining concepts from the data as extensively as possible (W. F. Wang & Cheng, 2025). At this stage, NVivo 12 was used to conduct line-by-line analysis of the original textual data. Vague or irrelevant statements were removed, and the interview transcripts were reviewed paragraph by paragraph to extract key elements reflecting young people’s experiences of beauty filter use. Through line-by-line analysis of the interview texts, a series of initial concepts were identified, covering the frequency, scenarios, intensity, function selection, purposes, and psychological feelings associated with beauty filter use. To preserve the originality and authenticity of the data, participants’ own words were used wherever possible during coding, ensuring that concept extraction was both accurate and representative. After repeated coding and discussion, 48 initial concepts were identified from the raw data. To make these concepts more organized and understandable, the 48 initial concepts were further grouped into 14 categories according to their core features and interrelationships, as shown in Table 2. These categories covered use frequency, use scenarios, self-presentation motivation, emotional needs motivation, social comparison psychology, ambivalence, and other dimensions. Table 2 presents only selected categories, concepts, and representative statements from the open coding, while the complete open-coding results are provided in the Supplementary Materials.

### 4.3. Axial Coding

Axial coding is a key stage in grounded theory analysis that further integrates and classifies the categories generated through open coding. Its core purpose is to identify internal connections and shared dimensions among categories and to build a more generalized system of core categories (H. Gu et al., 2025). Based on the 14 categories, this study further aggregated them into four core categories through axial coding. Categories reflecting regularity of use and dependence-related features were integrated into the core category of Beauty Filter Use Habits. Categories indicating tendencies in technology selection were grouped under the core category of Beauty Filter Use Preferences. Categories related to use purposes, emotional needs, and social expectations were integrated into the core category of Beauty Filter Use Motivations. Categories involving psychological responses during use were summarized as the core category of Psychological Responses to Beauty Filter Use, as shown in Table 3.

The axial coding results show that beauty filter use is jointly constituted by four core themes: Beauty Filter Use Habits, Beauty Filter Use Preferences, Beauty Filter Use Motivations, and Psychological Responses to Beauty Filter Use, which unfold across everyday image production, aesthetic choices, social expectations, and self-evaluation. Regarding Beauty Filter Use Habits, some participants had incorporated beauty filters or color adjustment into their routine process before image posting; *as M02 stated, “Even if I do not retouch my face, I will still adjust the color tone.” However, M12’s statement that “I still would not post bare-faced photos of myself on platforms”* also suggests that beauty filter use remains regulated by posting contexts and boundaries of self-presentation. Beauty Filter Use Preferences were reflected in choices of functions, tools, and visual styles. *M04 understood filters as “something aesthetic” and emphasized that “they are not about adjusting the portrait, but about adjusting the overall filter”; M16’s description of AI-generated group photos as “breaking the boundary between the two-dimensional and three-dimensional worlds”* also indicates that, for some users, beauty filters carry meanings of visual experimentation and technological playfulness. Beauty Filter Use Motivations further connected technological retouching with real-life self-adjustment, *as M18 described beauty filter results as “a kind of encouragement for my real self.” At the level of Psychological Responses to Beauty Filter Use, M20’s statement that “I am not very willing to let others know the real me”* reveals privacy boundaries and self-protection in digital image display. Thus, the relationship between beauty filters and self-image cognition is not limited to changes in appearance presentation; rather, it reflects users’ ongoing judgments among technological retouching, the real self, platform display, and others’ gaze.

### 4.4. Selective Coding

Selective coding aims to refine a core category from the main categories generated through axial coding and clarify the relational structure among them. This study identified “beauty filter use” as the core category and further examined how Beauty Filter Use Habits, Beauty Filter Use Preferences, Beauty Filter Use Motivations, and Psychological Responses to Beauty Filter Use relate to self-image cognition among Chinese youth. Specifically, Beauty Filter Use Motivations reflect individuals’ purposive reasons for using beauty filters and are linked to their expectations, evaluations, and presentation goals regarding their own image. Beauty Filter Use Habits indicate stable operational patterns formed through repeated use. Beauty Filter Use Preferences capture differentiated tendencies in the selection of functions, styles, and tools. Psychological Responses to Beauty Filter Use run through the use process in forms such as social comparison, defensive psychology, and ambivalence, and interact with subsequent experiences and self-evaluation. The interview materials show that the involvement of beauty filters in young people’s self-image is not merely a matter of psychological feeling or tool operation but involves both upstream cognitive processing and subsequent behavioral practice. Before and after using beauty filters, young people continuously assess self-presentation goals, appearance value, anticipated feedback from others, and social comparison, while linking these assessments to specific media use patterns and real-life image management behaviors. Thus, beauty filter use is not simply an act of image retouching, but a process of self-image adjustment shaped by the interplay between cognitive processing and behavioral practice.

**Figure 1:**
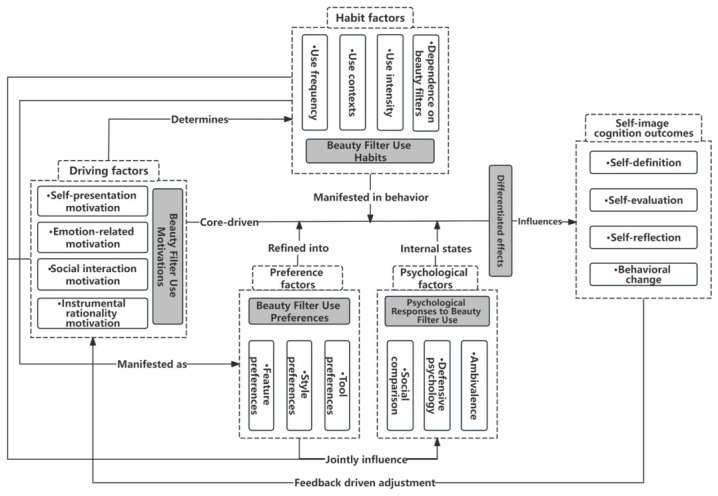
Dynamic Mechanism Model of Self-Image Cognition and Related Behavioral Responses in the Context of Digital Beauty Filter Use.

Conceptually, cognition mainly refers to individuals’ processes of perceiving, understanding, judging, and processing information about the self, others, and social contexts (Beer & Ochsner, 2006). Behavior, by contrast, mainly refers to individuals’ actions or inactions in response to internal or external stimuli (Levitis et al., 2009). The Theory of Planned Behavior suggests that psychological judgments such as attitudes, subjective norms, and perceived behavioral control are important conditions for understanding behavioral intentions and overt behaviors (Ajzen, 1991). Media effects research also emphasizes the need to distinguish cognitive and emotional responses from subsequent behavioral outcomes in media use (Valkenburg & Peter, 2013). Based on existing theoretical definitions and the interview findings, this study classifies Beauty Filter Use Motivations and Psychological Responses to Beauty Filter Use as cognitive conditions, and Beauty Filter Use Habits and Beauty Filter Use Preferences as behavioral conditions. Accordingly, this study does not conflate self-image cognition and behavioral responses into a single variable. Instead, it understands beauty filter use as a complex practice jointly constituted by cognitive and behavioral conditions. Together, these conditions form a dynamic mechanism that connects self-image cognition with related behavioral responses in the context of digital beauty filter use, as shown in Figure 1.

Beauty Filter Use Motivations constitute the cognitive starting point of young people’s self-projection, including motives for self-presentation, emotional needs, social interaction, and instrumental rationality. Through repeated practice, these motives become consolidated into a digitized bodily routine of “editing before posting,” blurring the cognitive boundary between the offline self and the digital self. Young people seek to balance social recognition and authenticity through personalized Beauty Filter Use Preferences and use beauty filters to expand the possibilities of self-presentation. Social comparison, defensive psychology, and ambivalence reflect the ongoing and dynamic negotiation of self-cognition under technological intervention. Individuals maintain a positive digital self while experiencing tension between dependence on technology and vigilance toward potential alienation. Overall, beauty filter use forms a dynamic mechanism that connects young people’s self-image cognition with related behavioral responses, thereby providing a qualitative basis for the subsequent fsQCA analysis.

### 4.5. Saturation Test

To assess theoretical saturation, this study reserved five interview transcripts that were not used in the earlier stages of open, axial, and selective coding. In the saturation test, the reserved transcripts were recoded line by line, concepts were re-extracted, and the results were compared with the established concepts, categories, and core categories. The results showed that all information in the reserved transcripts could be accommodated within the existing theoretical framework, with no new concepts or categories identified, indicating that the theoretical framework had reached saturation (Saunders et al., 2018).

## 5. Fsqca Analysis

### 5.1. Questionnaire Design and Data Collection

After completing the first-stage grounded theory analysis, this study transformed the core categories generated through qualitative coding into measurable antecedent conditions for the fsQCA stage and designed the questionnaire accordingly. The questionnaire included a study description and confidentiality statement, demographic information, confirmation of beauty filter use experience, and measurement items for each construct. All items were measured on a five-point Likert scale (1 = “strongly disagree,” 5 = “strongly agree”). In measuring the antecedent conditions, this study did not reduce beauty filter use to a single indicator of use frequency. Instead, it operationalized beauty filter use into four conditions: Beauty Filter Use Habits, Beauty Filter Use Preferences, Beauty Filter Use Motivations, and Psychological Responses to Beauty Filter Use. Beauty Filter Use Habits reflect use frequency, intensity, and dependence. Beauty Filter Use Preferences reflect tendencies in the selection of features, styles, and tools. Beauty Filter Use Motivations reflect purposive reasons such as self-presentation, social interaction, emotional regulation, and instrumental rationality. Psychological Responses to Beauty Filter Use reflect social comparison, image defense, and ambivalent feelings. Thus, the measurement of beauty filter use in this study covers behavioral, preference-related, motivational, and psychological dimensions, rather than merely examining whether beauty filters are used or how frequently they are used. The measurement items for each variable were mainly adapted from validated scales and contextualized for beauty filter use. Beauty Filter Use Habits were measured with reference to the SRBAI (Gardner et al., 2012), while also drawing on the SRHI’s measurement logic concerning behavioral stability and routinization (Verplanken & Orbell, 2003). Beauty Filter Use Motivations were measured with reference to the MSMU (Rodgers et al., 2021) and further adapted from the self-documentation and instrumental use dimensions of the social network use motivation scale (Masciantonio & Bourguignon, 2023). Psychological Responses to Beauty Filter Use were adapted from the ASMC to measure appearance comparison, evaluation concerns, and self-monitoring experiences (Choukas-Bradley et al., 2020). Beauty Filter Use Preferences were adapted from the PMS and its revised version (Gioia et al., 2023; McLean et al., 2015), focusing on tendencies in the use of features, styles, and retouching tools. Regarding outcome-variable specification, this study sets “self-image cognition” as the direct outcome variable in the fsQCA stage and treats related behavioral responses as an important extension of qualitative interpretation. In existing research, self-image cognition is often used to describe how individuals perceive, compare, and attach meaning to their own image (Ma et al., 2023). Meanwhile, research on self-image understands it as how individuals view themselves and their image, involving attitudes, views, and ideals related to the self (Simmons et al., 1973). In the context of digital beauty filter use, this study defines “self-image cognition” as the process through which individuals understand, evaluate, and confirm their appearance features, overall image value, and self-image stability during beauty filter use. This concept is related to, but not equivalent to, body image and self-concept clarity. Body image mainly emphasizes individuals’ evaluations, affective attitudes, and satisfaction with their physical appearance (Cash, 2004), whereas self-concept clarity emphasizes the clarity, consistency, and stability of individuals’ self-knowledge (Campbell et al., 1996). This study focuses on how, after the intervention of beauty filters, young people form judgments about their appearance, overall image, and self-stability across real, retouched, and ideal images, and how these judgments further relate to their media use and real-life behavioral responses. Therefore, in the questionnaire design stage, the measurement items for self-image cognition were adapted from the Appearance and Attribution dimensions of the BESAA and relevant items from the SCC scale, while being contextualized for beauty filter use. Specifically, the relevant BESAA dimensions were used to measure appearance self-evaluation and appearance attribution (Mendelson et al., 2001), while relevant SCC items were used to measure the clarity and stability of self-concept (Campbell et al., 1996). Related behavioral responses were mainly interpreted through the grounded theory materials. In the pilot survey, 100 Chinese youth participants completed the questionnaire. The results showed that the Cronbach’s α coefficients for all variables exceeded 0.8, with an overall scale reliability of 0.929, indicating high questionnaire reliability.

The formal survey was conducted through the Wenjuanxing platform from December 2025 to January 2026. The sample size was estimated using the Tests for One Coefficient Alpha procedure for a single Cronbach’s α coefficient. In PASS 2025, α was set at 0.05 and 1 − β at 0.90; considering 37 items, a null-hypothesis reliability coefficient of 0.90, an expected reliability coefficient of 0.92, and a 10% attrition rate, the minimum sample size was determined to be 353 (Bonett, 2002). The questionnaire was distributed using snowball sampling and promoted through QQ, WeChat, Xiaohongshu, and other platforms. Screening questions on birth year and beauty filter use experience were included. Only respondents born between 1981 and 2007 who had used digital beauty filters, photo-editing software, or related image-retouching tools were retained. To ensure data quality, attention-check items were included, and questionnaires with missing responses, completion times under 90 s, or obvious logical inconsistencies were excluded. A total of 693 questionnaires were collected, of which 635 were valid, yielding an effective response rate of 91.6% and meeting the minimum sample size requirement. The demographic characteristics of the sample are presented in Table 4.

### 5.2. Reliability and Validity Assessment

Before conducting the configurational analysis, this study first examined scale reliability. Cronbach’s α coefficients were calculated using SPSS 27.0, and the results are shown in Table 5. The Cronbach’s α coefficients for all variables exceeded 0.9, and the overall Cronbach’s α coefficient of the scale was 0.938, indicating high internal consistency.

Regarding validity testing, this study used SPSS 27.0 and AMOS 24.0 to assess convergent validity and discriminant validity. First, KMO and Bartlett’s test of sphericity were conducted in SPSS 27.0. As shown in Table 6, the KMO value was 0.922, and Bartlett’s test of sphericity was significant, indicating that the sample data were suitable for factor analysis.

Subsequently, confirmatory factor analysis (CFA) was conducted using AMOS 24.0. As shown in Table 7, the standardized factor loadings of all observed variables exceeded 0.5, the composite reliability (CR) values were all above 0.9, and the average variance extracted (AVE) values were all above 0.6 (Gan et al., 2025), indicating good convergent validity.

In addition, the discriminant validity results, shown in Table 8, indicate that the square root of the AVE for each factor was greater than its correlations with other factors (Fornell & Larcker, 1981), demonstrating good discriminant validity.

### 5.3. Common Method Bias Test

Harman’s single-factor test was used to assess common method bias in the collected sample data. An unrotated principal component factor analysis was conducted on all measurement items, and five factors with eigenvalues greater than 1 were extracted. The first factor explained 30.9% of the variance, which was below the 40% threshold (X. Chen et al., 2025), indicating that serious common method bias was not present in this study.

### 5.4. Correlation Analysis

This study used SPSS 26.0 and Pearson’s correlation coefficient (r) to conduct correlation analysis among the variables, and the results are shown in Table 9.

The correlation analysis results show significant positive correlations among all variables, with correlation coefficients ranging from 0.231 to 0.403. Among them, the correlation between Psychological Responses to Beauty Filter Use and Beauty Filter Use Habits was the highest, indicating a relatively strong empirical association between routinized use and appearance comparison, image defense, and ambivalent psychological experiences. Overall, although the variables were interrelated, the correlation coefficients did not reach a high level, suggesting that the different conditions were not simply substitutable. Therefore, it is necessary to further examine the configurational relationships between beauty filter use-related conditions and self-image cognition from the perspective of condition combinations.

### 5.5. Variable Selection and Calibration

Before conducting the fsQCA analysis, the raw scores of each variable needed to be calibrated into set membership scores ranging from 0 to 1. Common calibration methods include the indirect and direct methods. The direct calibration method converts continuous data into fuzzy-set membership scores using the Calibrate(x, n1, n2, n3) function in fsQCA 4.1 software, based on three anchors: full membership, the crossover point, and full non-membership. This method has strong replicability and has therefore been widely used in configurational research. In this study, self-image cognition was set as the outcome variable in the fsQCA analysis, while Beauty Filter Use Habits, Beauty Filter Use Preferences, Beauty Filter Use Motivations, and Psychological Responses to Beauty Filter Use were treated as the four antecedent conditions. Related behavioral responses were used as an important interpretive extension for understanding different states of self-image cognition. The earlier qualitative interviews showed that the relationship between beauty filter use, youth self-image cognition, and behavioral responses was not unidirectional. Some participants were able to incorporate beauty filters into a controllable process of self-presentation, using them as an auxiliary resource for understanding and adjusting self-image, which was associated with positive behavioral responses. Other participants, however, gradually treated technologically generated images as an important reference for evaluating their real selves during continuous use, showing denial of their real image or dependence on virtual images, which was even associated with negative behavioral responses. Therefore, this study examines not only the configurational pathways leading to high self-image cognition but also the condition combinations associated with non-high self-image cognition. Because fsQCA emphasizes causal asymmetry, high-level and non-high-level outcomes do not necessarily correspond to reverse combinations of the same conditions. Therefore, analyzing these two outcome states separately helps reveal the complex associations among different condition combinations, states of self-image cognition, and related behavioral responses in the context of digital beauty filter use.

Regarding variable calibration, there are currently no widely accepted clinical cutoffs or industry standards for Beauty Filter Use Habits, Beauty Filter Use Preferences, Beauty Filter Use Motivations, Psychological Responses to Beauty Filter Use, or Self-Image Cognition. Therefore, this study set calibration anchors based on the sample distribution. Specifically, n1, representing full membership, was set at the 95th percentile of each variable (x), indicating that a case had high membership in a given condition or outcome set; n2, representing the crossover point, was set at the 50th percentile, indicating maximum ambiguity between membership and non-membership; and n3, representing full non-membership, was set at the 5th percentile, indicating that a case had little or no membership in the set (Veríssimo, 2018). This procedure did not simply dichotomize the variables into “high” and “low” categories; rather, it used fuzzy-set membership scores to capture the relative degree of membership of each case in the relevant constructs. During calibration, the transformation was completed using fsQCA software. Following Fiss’s recommendation, all membership scores exactly equal to 0.5 were adjusted to 0.501 to prevent these cases from being automatically excluded from subsequent analysis (Fiss, 2011). In this study, “high level” does not merely refer to a high scale score but to high membership in the corresponding fuzzy set. Specifically, high Beauty Filter Use Habits indicate that an individual’s use of beauty filters is highly repetitive, routinized, and proceduralized. High Beauty Filter Use Preferences indicate that an individual has relatively clear and stable aesthetic orientations regarding filter styles, retouching methods, and beautification effects. High Beauty Filter Use Motivations indicate that an individual can clearly understand the instrumental or expressive functions of beauty filters in specific contexts. High Psychological Responses to Beauty Filter Use indicate that an individual has clear psychological perceptions and a relatively stable state of self-evaluation when facing beauty filters and the images they generate. High Self-Image Cognition indicates that an individual has a high level of understanding, evaluation, and confirmation of their appearance, overall image, and self-concept stability. Correspondingly, non-high levels or the absence of a condition does not mean that the relevant psychological responses, motivations, habits, or preferences are entirely absent. Rather, they indicate that a case has relatively low membership in the corresponding high-level set, meaning that the relevant construct has not reached the strength, clarity, or stability required to constitute a key condition. Therefore, non-high Self-Image Cognition is not equivalent to negative self-evaluation in a clinical sense. Rather, it indicates that a case has relatively low membership in the high Self-Image Cognition set and has not reached the set criteria for a highly stable and clear understanding, evaluation, and confirmation of self-image.

### 5.6. Necessity Analysis

After data calibration, this study conducted necessary condition analysis to determine whether each condition variable constituted a necessary condition for high Self-Image Cognition and non-high Self-Image Cognition. In general, if the consistency level of a condition variable exceeds 0.9, the condition can be regarded as a necessary condition for the outcome (A. W. Gu et al., 2022). The results of the necessary condition analysis are presented in Table 10.

The consistency of all condition variables was below 0.9, indicating that no single condition constituted a necessary condition for either high Self-Image Cognition or non-high Self-Image Cognition. This result indicates that the relationship between beauty filter use and self-image cognition cannot be explained by any single condition alone, nor can it be understood solely in terms of the level of a particular variable or the characteristics of a specific user group. Instead, it should be further understood through the configurational synergy among multiple conditions. Therefore, it is necessary to further conduct configurational analysis to examine how different antecedent conditions are associated with differentiated self-image cognition outcomes at the configurational level.

### 5.7. Configurational Analysis

Configurational analysis aims to explore how multiple antecedent conditions jointly operate and are associated with a specific outcome (Bacq & Alt, 2018). The preceding necessary condition analysis showed that no single condition variable alone constituted a necessary condition for either high Self-Image Cognition or non-high Self-Image Cognition. To further reveal the sufficiency relationships between different condition combinations and outcome states, this study conducted configurational analysis by constructing a truth table. Following prior studies, this study set the consistency threshold at 0.8, the case-frequency threshold at 1 (Tao et al., 2021), and the PRI consistency threshold at 0.65 (T. Zhou et al., 2022). Based on these settings, the complex, intermediate, and parsimonious solutions for the conditions associated with self-image cognition among Chinese youth were calculated separately, as shown in Table 11.

As shown in Table 11, two equivalent configurational pathways were identified for high Self-Image Cognition, which can be summarized as the “psychological-response–motivation core coexistence” type and the “psychological-response–preference core coexistence” type. For non-high Self-Image Cognition, three configurational pathways were identified. Configurations 1 and 2 can be summarized as the “dual absence of psychological responses and motivations” type, while Configuration 3 can be characterized as the “motivational-core absence with coexisting habits and preferences” type. These results indicate that self-image cognition cannot be explained by any single condition alone but instead presents differentiated outcome states across different combinations of conditions. The pathways for high and non-high Self-Image Cognition are not simple reverse patterns, reflecting the configurational asymmetry emphasized by fsQCA.

#### 5.7.1. Configurations for High Self-Image Cognition

##### Psychological-Response–Motivation Core Coexistence Type (Configuration 1)

In this configuration, Psychological Responses to Beauty Filter Use and Beauty Filter Use Motivations are core presence conditions, Beauty Filter Use Preferences serve as a peripheral absence condition, and Beauty Filter Use Habits do not constitute a key condition. When Chinese youth use beauty filters with clear goals and relatively stable psychological states, beauty filter use remains associated with a higher level of Self-Image Cognition, even without fixed use habits or specific preferences. The key to this configuration is that clear use motivations make filtered images more likely to be understood as image adjustments for specific contexts, such as job seeking or further education, rather than as fixed standards for everyday self-evaluation. A relatively stable psychological state also makes individuals less likely to fall into intense social comparison or self-doubt, allowing them to distinguish instrumental presentation from the real self after retouching. Therefore, in this pathway, beauty filters function more as a situational supplement to confidence than as a substitute for the real self.

The interview materials further show that this configuration is not concentrated in any specific age group but reflects a relatively consistent instrumental understanding among participants of different ages. *F06 stated, “If I want to look more refined today, I will retouch the photo before posting it; but if I encounter something interesting in daily life, posting the original photo directly is also fine. It depends on the occasion and the situation.”* In this pathway, younger participants were more likely to decide whether to use beauty filters in specific contexts such as social media posting and life recording, while maintaining boundaries between real and retouched images. *M18 noted, “After beauty filters became available, everyone has paid more attention to appearance. I also do things like skincare, and it pushes me to become better.”* Although slightly older participants were also concerned with image presentation, their behavioral responses were more reflected in routinized and functional real-life maintenance, such as skincare and image management. Thus, although participants of different ages differed in their life contexts and observable behavioral expressions, the mechanism of this pathway lies in the fact that stable psychological responses and clear motivations jointly provide goal boundaries for beauty filter use. This enables individuals to avoid treating filtered images as substitutes for the real self; instead, they understand them as contextualized and instrumental means of image management, which are associated with adaptive behavioral responses such as moderate retouching, naturalized self-presentation, and everyday image maintenance.

##### Psychological-Response–Preference Core Coexistence Type (Configuration 2)

In this configuration, Psychological Responses to Beauty Filter Use and Beauty Filter Use Preferences are core presence conditions, Beauty Filter Use Habits are a core absence condition, and Beauty Filter Use Motivations do not constitute a key condition. In other words, when Chinese youth have relatively stable psychological responses and clear technological preferences, beauty filter use remains associated with a higher level of Self-Image Cognition, even in the absence of high-frequency use habits or explicit motivations. The key to this pathway does not lie in frequency of use, but in whether young people can maintain a stable psychological state and keep beauty filter use within aesthetic boundaries they recognize and accept.

This configuration was relatively more typical among participants in their 20s to around 30. Young people at this life stage are often in a relatively active period of identity exploration, aesthetic experimentation, and social expression, making technologically generated idealized images more likely to be incorporated into their style expression and self-presentation. *F02 stated, “I do not post very often. I only want to post at some relatively special moments, such as when I take a photo in which my mother and I both look good and the atmosphere feels loving.” She also noted, “For anything I post, I probably adjust it a little, mainly to the style I personally like.”* In this use experience, beauty filters do not function as a high-frequency dependence for maintaining confidence, but as stylistic adjustments serving specific contexts and personal aesthetics. Individuals are able to understand technologically generated idealized images within aesthetic boundaries they recognize and maintain judgment about the relationship between the real self and the retouched image.

Further, this higher level of Self-Image Cognition may also be connected to real-life style learning and image adjustment. *F14 stated, “Some time ago, I edited myself with a very upturned eyeliner. Others said that the eyeliner I edited looked really good, so in real life I immediately drew that eyeliner. It pushed me in the opposite direction to do something more positive for myself and make a better change in my image.”* In this process, the idealized image generated by beauty filters does not replace the real self but becomes a reference for real-life makeup learning and image optimization. Therefore, the mechanism of this pathway lies in the fact that clear preferences and stable psychological responses jointly define the aesthetic boundaries of beauty filter use among Chinese youth. In this way, beauty filters function more as tools for aesthetic calibration and style reference than as a one-way dependence on virtual images and are associated with adaptive behavioral responses such as style expression, makeup learning, and real-life image adjustment.

#### 5.7.2. Configurations for Non-High Self-Image Cognition

##### Dual Absence of Psychological Responses and Motivations Type (Configurations 1 and 2)

In this category, although Configurations 1 and 2 differ in behavioral conditions, both have Psychological Responses to Beauty Filter Use and Beauty Filter Use Motivations as core absence conditions; therefore, they can be summarized as the “dual absence of psychological responses and motivations” type. Specifically, Configuration 1 has Beauty Filter Use Habits as a core presence condition, while Beauty Filter Use Preferences does not constitute a key condition. Configuration 2, by contrast, has Beauty Filter Use Preferences as a core presence condition, while Beauty Filter Use Habits does not constitute a key condition. In other words, when Chinese youth lack relatively stable psychological support and clear use motivations when using beauty filters, beauty filter use remains associated with non-high Self-Image Cognition, even if they have developed fixed use habits or aesthetic preferences. The key does not lie in whether use is frequent or preferences are clear, but in the lack of goal boundaries and psychological regulation during the use process. Under such conditions, some young people may become more reliant on technologically generated images to evaluate their real selves through repeated retouching, and may come to regard original photos, bare-faced photos, or natural states as images unsuitable for public display.

This pathway was relatively more concentrated in the narratives of younger participants who were more active on social media. These young people are at a stage characterized by active self-identity construction, heightened aesthetic perception, and greater social visibility, making them more likely to develop concerns about their real images through peer feedback, platform-based aesthetics, and comparisons with idealized images. When individuals lack stable psychological support and clear use motivations, technologies originally used to modify images are more likely to enter the process of real-self evaluation and become associated with avoidant behavioral responses.

The pathway with Beauty Filter Use Habits as a core presence condition more strongly highlights the link between default retouching and unease about the real image. *F01 mentioned, “Once I posted a photo without removing my nasolabial folds. Later, the more I looked at it, the more uncomfortable I felt. Although people had already liked and commented on it, I still deleted it because I felt that it was not a photo that could represent my state.”* In this type of experience, once retouching becomes a routine step before posting, some young people’s acceptance of unedited images declines. The real image may then be regarded as a version insufficient to represent the self, while subsequent behaviors take the form of immediate corrective responses, such as re-retouching photos or deleting display materials that do not meet expectations.

The pathway with Beauty Filter Use Preferences as a core presence condition more strongly highlights the link between fixed style preferences and the standardization of retouched images. *F14 stated, “If I had to post the original photo without using beauty filters, I would feel particularly anxious. I usually only keep the retouched one, because I would rather keep that better-looking version of myself in my album and in front of others.”* As fixed preferences are repeatedly reinforced, the retouched image gradually becomes the standard version more suitable for preservation and display, while the real image is placed in a position that individuals are unwilling to keep or face. Thus, the mechanism of this pathway lies in the fact that when stable psychological responses and clear motivations are both absent, existing use habits or aesthetic preferences can hardly form effective self-regulatory boundaries. Images generated by beauty filters are therefore more likely to enter Chinese youth’s evaluation of the real self and become associated with avoidant behavioral responses such as avoidance of original images, repeated retouching, and deletion of display materials that do not meet expectations, thereby compressing young people’s space for authentic self-presentation.

##### Motivational-Core Absence with Coexisting Habits and Preferences Type (Configuration 3)

In this configuration, Beauty Filter Use Motivations are a core absence condition, Beauty Filter Use Habits are a core presence condition, Beauty Filter Use Preferences are a peripheral presence condition, and Psychological Responses to Beauty Filter Use do not constitute a key condition. When Chinese youth lack clear and stable use motivations but have developed a high level of use habits along with certain retouching preferences, beauty filter use is more likely to become default and proceduralized. In this situation, young people do not necessarily retouch images to complete a specific act of self-presentation; rather, through repeated operation, they come to treat “retouching before posting” as a basic procedure for public expression. Over time, technologically processed images are more likely to be understood as the standard version suitable for being seen, accepted, and posted, while real appearance is placed in a relatively secondary position within proceduralized retouching and becomes associated with non-high Self-Image Cognition.

This pathway was relatively more evident in the narratives of slightly older participants with more stable social roles. Compared with younger participants, who more often engaged in style exploration, social feedback, and immediate image adjustment, these participants’ image management was more embedded in occupational roles, family roles, and everyday social relationships. Their related behavioral responses were also more likely to settle into routinized and functional processes of image maintenance. *F24 stated, “I must use beauty filters when posting photos; otherwise, I feel very uncomfortable. Basically, after retouching, I directly synchronize the photo to WeChat and Douyin with one click, and I do not adjust it again for different platforms.” M21 also noted, “Beauty filtering is a fixed workflow. It is not that I have to retouch the image in an exaggerated way, but I always adjust the parameters a little. Now it feels more like a necessary step, and I do not really think about whether to do it anymore.”* These experiences suggest that this pathway does not necessarily manifest as immediate appearance anxiety or strong behavioral impulses but is more likely to appear over long-term repetition as a low-reflection standard for public self-presentation. At this point, although preferences serve only as a peripheral presence condition, they are still linked to fixed parameters and templated presentation, making individuals tend to complete image posting according to established routines.

Overall, the key to this pathway does not lie in whether young people have a strong need for beauty filters, but in the fact that, when clear motivations are absent, existing use habits and certain preferences jointly push beauty filters from contextualized retouching tools into a default, proceduralized, and templated posting mechanism. Compared with the previous two pathways of non-high Self-Image Cognition, the risk of this pathway is not mainly manifested as immediate anxiety about original images or strong denial of specific flaws. Rather, it is manifested in individuals’ continued use of fixed retouching procedures in a low-reflection state, which gradually weakens their ability to distinguish between real appearance and online presentation. Therefore, this pathway is more likely to be associated with behavioral responses such as default retouching, template repetition, and the continued use of fixed parameters, making the relationship between young people’s real selves and online presentation somewhat blurred.

### 5.8. Robustness Check

This study adopted a set-theoretic robustness test to examine the robustness of the configurational results. Keeping the case-frequency threshold and PRI threshold unchanged, the consistency threshold used in truth table screening was adjusted to 0.68 to test the reliability of the configurational results. The results showed that, after the threshold adjustment, the configurations for high Self-Image Cognition and non-high Self-Image Cognition remained unchanged compared with the original results. Therefore, the findings did not change substantially with the adjustment of the consistency threshold, indicating a high level of robustness.

## 6. Discussion

First, the grounded theory results show that the relationship between beauty filter use and self-image cognition among Chinese youth is not a simple accumulation of single retouching behaviors, but a technologically mediated relationship formed through everyday image production and social self-presentation. Beauty Filter Use Motivations, Beauty Filter Use Habits, Beauty Filter Use Preferences, and Psychological Responses to Beauty Filter Use jointly participate, through repeated use, in the process through which young people understand, view, and evaluate the self. As “retouching before posting” gradually becomes a routine practice, the technologically processed online image is no longer merely an attachment to the real image but becomes one important reference through which young people judge whether they look attractive, appropriate, and suitable to be seen. Accordingly, beauty filter use is associated not only with changes in image appearance but also with adjustments in the way self-image cognition is formed and further connects with related behavioral responses in real life. This reveals a mechanism through which technological retouching enters self-evaluation and becomes associated with behavioral responses.

Second, the fsQCA results further reveal the nonlinear configurational relationship between beauty filter use and self-image cognition. The relationship between beauty filter use and self-image cognition is not a single linear relationship but is characterized by differentiated combinations of conditions and outcome states. The two pathways leading to high Self-Image Cognition show that when young people have relatively stable psychological states and either clear use motivations or clear technological preferences, beauty filters are more likely to be incorporated into controllable image expression. In this situation, technologically generated images do not replace judgments about the real self, but are associated with a sense of image coherence, control, and confirmation, and may further connect with real-life self-care behaviors such as skincare and fitness. Thus, high Self-Image Cognition does not depend on reducing or rejecting beauty filter use, but on whether individuals can maintain a switchable relationship between retouching and the real self, and understand technological feedback as a reference resource for self-optimization. This selective absorption of technological feedback and boundary-oriented processing also suggests that relatively healthy beauty filter use should be reflected in young people’s ability to confirm the real self and maintain boundary awareness regarding technologically generated images. The pathways of non-high Self-Image Cognition, by contrast, show more evident risk-related associations. When young people lack or chronically lack psychological support and use motivations, beauty filters are more likely to shift from contextualized tools of expression to routinized and standardized references for self-evaluation. Young people may then use technologically generated images to evaluate their real selves and regard unretouched appearance as insufficiently attractive or unsuitable for display. Behaviorally, this is more likely to manifest as avoidance of original images, increased retouching intensity, and dependence on refined retouched versions. If technologically generated images become a long-term reference for viewing the self, individuals may become more critical in evaluating their face shape, facial features, and skin texture, with potential links to appearance anxiety, self-doubt, and intentions to alter their real-life image.

Further, the relationship among beauty filter use, youth self-image cognition, and behavioral responses involves both common mechanisms across age stages and different manifestations shaped by life-stage differences. Overall, technologically generated retouched images enter, to varying degrees, individuals’ processes of understanding, evaluating, and confirming the real self. However, this relationship does not primarily depend on age itself, but on whether individuals can maintain clear goal boundaries, psychological regulation, and self-evaluation standards during beauty filter use. Age differences are more reflected in how this mechanism is manifested at the behavioral level. Younger youth are usually at a stage characterized by more active social expression, aesthetic experimentation, and platform interaction, making beauty filter use more likely to be connected with style exploration, makeup learning, and concerns about publicly displaying the real image. Older youth, by contrast, are more often at a stage in which occupational roles, family relationships, and everyday social relationships are relatively stable, making beauty filter use more likely to be embedded in real-life image maintenance and routine posting practices. Thus, age or life stage does not change the basic relational logic between beauty filter use and self-image cognition; rather, it is more reflected in the different ways this logic manifests in specific behavioral responses.

Overall, the cognitive and behavioral differences shown by Chinese youth in beauty filter use reflect changes in the relational positioning between the real subject-self and the virtual object-self. Beauty filters provide young people with convenient resources for image expression, but this process is not entirely neutral. When technological feedback enters the process of self-evaluation over time, the virtual object-self online may become an important reference through which young people understand, accept, and present the real subject-self. High Self-Image Cognition indicates that young people can still keep technology within the scope of auxiliary self-management and maintain the ability to confirm the real self. Non-high Self-Image Cognition, by contrast, indicates that the relationship between technologically generated images and judgments about the real self is more likely to become blurred and is further associated with risk-related behavioral responses such as avoidance of original images, dependence on retouching, and weakened real-life action. Through a mixed-methods approach, this study reveals the complex mechanisms of Chinese youth’s self-image cognition and related behavioral responses in the context of digital beauty filter use, providing empirical evidence for understanding how young people maintain a balance between technological convenience and self-authenticity in the digital visual era.

## 7. Conclusions

### 7.1. Theoretical Implications

The theoretical contribution of this study lies not only in revealing the complex mechanisms of Chinese youth’s self-image cognition and related behavioral responses in the context of digital beauty filter use but also in advancing this issue to the level of how subjectivity is reorganized after digital technologies become deeply embedded in everyday life. Beauty filters are not neutral tools that merely serve appearance presentation; rather, through continuous use, they participate in the processes through which young people understand the self, evaluate the self, and connect these evaluations with real-life behavioral responses. Once technologically generated displayable images repeatedly enter everyday self-confirmation, the ways individuals view their appearance, temperament, and overall image also become connected with the technological logic of modifiability, displayability, and comparability. Accordingly, discussions of beauty filters no longer remain at the level of image optimization or media effects but further point to how digital technologies participate in subjects’ confirmation of self-standards and their understanding of the boundary between the real self and the ideal self.

The theoretical significance of this study also lies in extending the cultural explanatory dimension of research on the relationship between beauty filters and self-image cognition. Western individualistic cultures place greater emphasis on individual choice and self-expression, and existing studies on filters, body image, and digital self-presentation have often focused on issues such as body satisfaction, self-acceptance, personal authenticity, and appearance anxiety. Within this cultural logic, the conflict between the real self and the ideal self is more often manifested in how individuals confront the gap between idealized images and the unretouched body. By contrast, the Chinese cultural context places greater emphasis on the relational self, others’ evaluations, and mianzi, or “face,” so individual image is not merely a private aesthetic choice but is often connected to social judgments about whether one appears appropriate, suitable to be seen, and likely to gain recognition. Therefore, the conflict between the real self and the ideal self faced by Chinese youth in beauty filter use is not simply a matter of body satisfaction but an ongoing negotiation among the real self, digitally retouched images, relational evaluation, and social expectations. Accordingly, this study extends related research beyond Western frameworks of individual psychology and body satisfaction and further situates it within an explanatory pathway in which relational evaluation, mianzi logic, and social competition pressures participate in digital self-construction in the Chinese cultural context.

Further, the relationship between digital technologies and subjects does not unfold in a single direction but shows clear conditional differences. Beauty filter use may be associated with real-life self-management behaviors such as image confirmation, skincare, fitness, and makeup management, but it may also be connected with risk-related behavioral responses such as insufficient acceptance of the real self, avoidance of original images, pressure to display one’s real appearance, and intensified beauty filter use. What truly deserves attention is no longer merely whether beauty filters are related to self-image cognition but how digital technologies participate, under different combinations of conditions, in subjects’ processes of constructing the self, evaluating the self, and acting in real life. This study extracted four core categories from the interview materials—motivations, habits, preferences, and psychological responses—and used fsQCA to show how different combinations of antecedent conditions are associated with differentiated states of self-image cognition. In doing so, it avoids reducing the relationship between technology and self-image cognition to a single variable, a single pathway, or a one-directional conclusion. It also provides a more contextualized explanatory perspective for understanding how, after digital technologies become deeply embedded in everyday life, people’s cognitive boundaries, modes of self-confirmation, and logics of real-life action are reorganized.

### 7.2. Practical Implications

From both individual and social dimensions, this study provides practical implications for guiding young people to use beauty filters more prudently, enhance psychological resilience, and develop adaptive patterns of media use. Digital beauty filters are associated not only with changes in aesthetic preferences but also with young people’s processes of self-understanding, self-presentation, and self-regulation. Therefore, in practice, the emphasis should not be placed simply on reducing or avoiding beauty filter use, but on helping young people establish a sense of boundaries among technological convenience, social expectations, and the real self. Young people need to recognize that a more refined and displayable image on platforms is not equivalent to the value of the real self. Retouched photos may serve expression in specific contexts, but they should not replace judgments about the real self. Healthy beauty filter use does not mean rejecting retouching, but retaining interpretive control over technologically generated images, the ability to confirm the real self, and psychological resilience when facing appearance comparison and platform evaluation, so that beauty filters can become tools for auxiliary expression and self-adjustment.

This issue is not an isolated problem at the individual level among young people, but is closely related to platform logic, educational environments, and social support systems. When designing beauty filter templates, default parameters, and recommendation logics, platforms should reduce the concentrated reinforcement of narrow aesthetic standards such as fair skin, slim faces, and youthfulness. They should also preserve users’ space for judging the real self through measures such as original-image comparisons, retouching reminders, and natural-style options. University mental health education, community youth services, and youth development work should also include phenomena such as excessive dependence on retouching and avoidance of real-appearance display within their scope of observation and support. Through media literacy education, awareness of body diversity, self-acceptance training, and psychological resilience cultivation, they can guide young people to understand the constructed nature and limitations of technologically generated images and to learn bounded, reflective, and selective self-presentation across different media contexts. Only when society as a whole attends to the complex associations among young people’s cognition, emotions, and behavioral responses in the context of digital beauty filter use can digital technologies more likely become resources for young people to expand expression, adjust the self, and connect with the world.

### 7.3. Limitations and Future Research

By combining grounded theory and fsQCA, this study reveals the complex associations between Chinese youth’s self-image cognition and related behavioral responses in the context of digital beauty filter use, but several limitations remain. First, both the interview materials and questionnaire data mainly relied on participants’ self-reports, which may be affected by subjective perceptions and recall bias. Meanwhile, the measurement of self-image cognition in this study was adapted from relevant dimensions of existing validated scales and contextualized for beauty filter use. Although the scale passed the pilot survey as well as reliability and validity tests, it remains different from an independent scale specifically developed for the digital beauty filter context. Future research may incorporate platform behavioral data, image-posting records, or experimental materials, and further develop measurement tools for self-image cognition with stronger contextual adaptability.

Second, this study adopted a cross-sectional research design. Although this design can reveal the configurational relationships between beauty filter use and self-image cognition, it remains limited in fully capturing their dynamic changes over time. Future studies may use longitudinal tracking, repeated interviews, or experimental designs to further examine the dynamic associations among beauty filter use, self-image cognition, and real-life behavioral responses. In addition, although this study included participants with different identities and occupational backgrounds, it did not systematically compare differences among students, working youth, and users in different occupational categories. Subsequent research may further incorporate social roles and life contexts into the analytical framework.

Finally, this study was conducted within the Chinese sociocultural context, and its findings are therefore strongly context-specific. Their applicability across different cultural backgrounds, platform environments, and media systems remains to be further tested. Future research may combine cross-cultural comparison, cross-platform comparison, and multi-method research designs to further test the robustness of the findings and extend understanding of the relationships among digital visual technologies, self-image cognition, and youth behavioral responses.

## Figures and Tables

**Table 1:** Participant characteristics.

ID	Gender	Age	Education	Occupation	Interview Mode	Interview Length (min)
M01	Male	20	Associate degree	Student	In-person	53
M02	Male	22	Bachelor’s degree	Student	Online	58
M03	Male	24	Bachelor’s degree	Student	In-person	47
M04	Male	25	Bachelor’s degree	Enterprise employee	Online	63
M05	Male	23	Bachelor’s degree	Student	In-person	55
M06	Male	27	Postgraduate	Public institution staff	Online	68
M07	Male	21	Associate degree	Student	In-person	50
M08	Male	28	Postgraduate	Enterprise employee	Online	62
M09	Male	30	Bachelor’s degree	Freelancer	In-person	57
M10	Male	26	Bachelor’s degree	Enterprise employee	Online	54
M11	Male	32	Postgraduate	Public institution staff	In-person	65
M12	Male	19	Associate degree	Student	Online	46
M13	Male	29	Bachelor’s degree	Freelancer	In-person	60
M14	Male	34	Postgraduate	Public institution staff	Online	70
M15	Male	24	Bachelor’s degree	Student	In-person	51
M16	Male	35	Bachelor’s degree	Enterprise employee	Online	59
M17	Male	31	Postgraduate	Freelancer	In-person	64
M18	Male	38	Bachelor’s degree	Enterprise employee	Online	66
M19	Male	41	Postgraduate	Public institution staff	In-person	69
M20	Male	33	Bachelor’s degree	Freelancer	Online	56
M21	Male	43	Associate degree	Freelancer	In-person	53
F01	Female	18	Associate degree	Student	Online	45
F02	Female	20	Bachelor’s degree	Student	In-person	50
F03	Female	22	Bachelor’s degree	Student	Online	55
F04	Female	21	Associate degree	Student	In-person	48
F05	Female	23	Bachelor’s degree	Student	Online	52
F06	Female	24	Bachelor’s degree	Student	In-person	58
F07	Female	25	Postgraduate	Student	Online	61
F08	Female	26	Bachelor’s degree	Enterprise employee	In-person	64
F09	Female	27	Postgraduate	Enterprise employee	Online	59
F10	Female	28	Bachelor’s degree	Public institution staff	In-person	62
F11	Female	22	Bachelor’s degree	Student	Online	54
F12	Female	29	Postgraduate	Public institution staff	In-person	67
F13	Female	24	Bachelor’s degree	Student	Online	53
F14	Female	30	Postgraduate	Enterprise employee	In-person	66
F15	Female	31	Bachelor’s degree	Freelancer	Online	60
F16	Female	32	Postgraduate	Public institution staff	In-person	68
F17	Female	33	Bachelor’s degree	Freelancer	Online	58
F18	Female	34	Postgraduate	Enterprise employee	In-person	65
F19	Female	35	Bachelor’s degree	Freelancer	Online	63
F20	Female	36	Postgraduate	Public institution staff	In-person	70
F21	Female	38	Bachelor’s degree	Enterprise employee	Online	62
F22	Female	40	Postgraduate	Freelancer	In-person	69
F23	Female	42	Bachelor’s degree	Public institution staff	Online	65
F24	Female	44	Postgraduate	Public institution staff	Online	70

**Table 2:** Examples of open coding (excerpt).

Category	Concept	Raw Interview Excerpt (Participant ID)
A1 Use frequency	a1 High-frequency use	“I do it every time. With the original camera, I usually prefer a more retro tone, the kind with stronger contrast. Even if I do not retouch my face, I still adjust the tone to match the feel I like.” (M02)
	a2 Moderate-frequency use	“Unedited photos are usually landscapes, or some cute shots that are not of people. If it is a person, I usually do a bit of retouching.” (F08)
	a3 Low-frequency use	“Not very often, unless the school requires us to post group photos of students. I might post when I travel, but I still would not post personal photos on the platform.” (M12)
A2 Use contexts	a4 Platform context	“Definitely, because I think social media itself carries this desire to share.” (F07)
	a5 Content context	“If it is a person, I will do a bit of retouching. Even if it is not me and it is my friend posting it, I will still beautify it a little. I think it is a form of respect, respect for them.” (F08)
	a6 Social context	“What moves me most is social relationships, because people are not isolated individuals; it is about showing the good in oneself. For me, photos that show close relationships, such as taking a group photo together, and then beautifying it a bit, are more moving.” (F12)
…	…	…
A8 Self-presentation motivation	a23 Idealized self-presentation	“On social platforms, after beautification, what is presented is an idealized version of oneself.” (F02)
	a24 Meeting presentation needs	“On social platforms you definitely want to show a better self, to build a so-called persona, so I will choose photos that have been retouched before posting.” (F05)
	a25 Ritualized life recording	“I share it partly to record my life, because I am someone who really likes posting to Moments. Every time I post, the photos have to look perfect.” (F06)
…	…	…
A12 Social comparison psychology	a39 Upward comparison	“That person is genuinely very good-looking, from bone structure to skin. Even if I edit my photo, it still does not look as good as their original, and that makes me feel a bit of appearance anxiety.” (F15)
	a40 Lateral comparison	“In everyday life, for example, my roommate has very fair skin. I often say, you are so white, and then I ask her to stretch out her arm. I am like an Oreo, the cream filling. I feel like the outer shell is real, and then you also think, why is my skin tone so dark?” (F20)
		
Total: 14 categories	Total: 48 concepts	

Note. The table presents an excerpt of open-coding results based on participants’ verbatim interview excerpts; category and concept labels were inductively generated from the data.

**Table 3:** Results of axial coding.

Core Category	Category	Category Description
Beauty Filter Use Habits	Use frequency	The distribution and regularity of how often beauty filters are used.
	Use contexts	The set of specific contexts in which beauty filters are used, including platform type, content type, and social circles.
	Use intensity	The degree to which beauty filters are used to modify one’s appearance, ranging from light to moderate to heavy modification.
	Dependence on beauty filters	The level of reliance and stickiness in using beauty filters when posting appearance-related content, including high, moderate, and low dependence.
Beauty Filter Use Preferences	Feature preferences	Prioritized choices among feature types when using beauty filters, including basic retouching, advanced reshaping, and AI-generated functions.
	Style preferences	Preferred directions and emphases among filter styles when enhancing appearance, such as natural, atmospheric, and refined styles.
	Tool preferences	Preferences and priorities across tool types used for beautification, including dedicated beauty-camera apps, professional editing software, and AI-based beauty tools.
Beauty Filter Use Motivations	Self-presentation motivation	Purposive reasons for using beauty filters to achieve self-expression goals, such as idealized self-presentation and recording life moments.
	Emotion-related motivation	Emotion-oriented motives for using beauty filters to meet emotional needs, such as gaining others’ approval, alleviating appearance anxiety, or compensating for perceived image flaws.
	Social interaction motivation	Social-feedback-oriented motives for using beauty filters to increase social media likes, comment interactions, and other forms of social feedback.
	Instrumental rationality motivation	Instrumental reasons for using beauty filters to meet practical needs, such as correcting lens distortion and optimizing image color, lighting, and visual texture.
Psychological Responses to Beauty Filter Use	Social comparison	Psychological states arising during beauty filters use, including comparisons with others’ filtered images, benchmarking against one’s ideal image, and efforts to avoid negative comparisons.
	Defensive psychology	Defensive tendencies formed after using beauty filters to manage potential risks, such as coping with negative evaluations or concealing perceived flaws.
	Ambivalence	Ambivalent psychological states at the cognitive and behavioral levels, shaped by dependence on filter effects alongside vigilance toward possible cognitive distortion.

Note. Core categories and subcategories were developed through axial coding of the interview data.

**Table 4:** Sample characteristics.

Item	Category	*n*	%
Gender	Male	319	50.2
	Female	316	49.8
	18–24	286	45.0
	25–29	105	16.5
Age (years)	30–35	96	15.1
	36–41	90	14.2
	42–44	58	9.1
	High school or below	74	11.7
Education	Associate degree	91	14.3
	Bachelor’s degree	265	41.7
	Postgraduate or above	205	32.3
Long-term city of residence/work	Tier 1 city	156	24.6
	New Tier 1 city	185	29.1
	Tier 2 city	162	25.5
	Tier 3 or below	132	20.8
Occupation/status	Student	235	37.0
	Enterprise employee	150	23.6
	Public institution staff	135	21.3
	Freelancer	115	18.1

Note. *N* = 635. Percentages may not sum to 100 due to rounding.

**Table 5:** Reliability results.

Variable	No. of Items	Cronbach’s α
Beauty Filter Use Habits	7	0.924
Beauty Filter Use Motivations	8	0.936
Psychological Responses to Beauty Filter Use	7	0.929
Beauty Filter Use Preferences	7	0.927
Self-Image Cognition	8	0.939
Overall scale	37	0.938

Note. α denotes Cronbach’s alpha. The overall scale includes all 37 items.

**Table 6:** KMO and Bartlett’s test of sphericity.

KMO Measure of Sampling Adequacy		0.922
Bartlett’s test of sphericity	Approx. chi-square	17,023.421
	df	666
	Sig.	<0.001

Note. KMO values above 0.80 indicate adequate sampling; a significant Bartlett’s test suggests the data are suitable for factor analysis.

**Table 7:** Convergent validity results.

Latent Construct	Indicator	Standardized Loading	AVE	CR
Beauty Filter Use Habits	Habit1	0.797	0.634	0.924
	Habit2	0.814		
	Habit3	0.803		
	Habit4	0.819		
	Habit5	0.756		
	Habit6	0.797		
	Habit7	0.787		
Beauty Filter Use Motivations	Motivation1	0.811	0.648	0.936
	Motivation2	0.808		
	Motivation3	0.805		
	Motivation4	0.819		
	Motivation5	0.793		
	Motivation6	0.805		
	Motivation7	0.790		
	Motivation8	0.806		
Psychological Responses to Beauty Filter Use	Psychological Response1	0.795	0.652	0.929
	Psychological Response2	0.796		
	Psychological Response3	0.812		
	Psychological Response4	0.821		
	Psychological Response5	0.809		
	Psychological Response6	0.811		
	Psychological Response7	0.807		
Beauty Filter Use Preferences	Preference1	0.806	0.645	0.927
	Preference2	0.795		
	Preference3	0.825		
	Preference4	0.818		
	Preference5	0.770		
	Preference6	0.802		
	Preference7	0.803		
Self-Image Cognition	Self-Image Cognition1	0.809	0.661	0.940
	Self-Image Cognition2	0.813		
	Self-Image Cognition3	0.800		
	Self-Image Cognition4	0.811		
	Self-Image Cognition5	0.841		
	Self-Image Cognition6	0.784		
	Self-Image Cognition7	0.797		
	Self-Image Cognition8	0.845		

Note. AVE = average variance extracted; CR = composite reliability. Loadings are standardized.

**Table 8:** Discriminant validity results.

	Self-Image Cognition	Psychological Responses to Beauty Filter Use	Beauty Filter Use Preferences	Beauty Filter Use Motivations	Beauty Filter Use Habits
Self-Image Cognition	0.813				
Psychological Responses to Beauty Filter Use	0.366	0.803			
Beauty Filter Use Preferences	0.265	0.307	0.808		
Beauty Filter Use Motivations	0.333	0.248	0.365	0.805	
Beauty Filter Use Habits	0.252	0.435	0.336	0.346	0.796

Note. Diagonal values are square roots of AVE. Off-diagonal values are inter-construct correlations.

**Table 9:** Correlation Matrix of the Variables.

Variable	Beauty Filter Use Habits	Beauty Filter Use Motivations	Psychological Responses to Beauty Filter Use	Beauty Filter Use Preferences	Self-Image Cognition
Beauty Filter Use Habits	1				
Beauty Filter Use Motivations	0.323 ***	1			
Psychological Responses to Beauty Filter Use	0.403 ***	0.231 ***	1		
Beauty Filter Use Preferences	0.313 ***	0.343 ***	0.287 ***	1	
Self-Image Cognition	0.236 ***	0.314 ***	0.343 ***	0.248 ***	1

Note. *N* = 635; *** *p* < 0.001.

**Table 10:** Necessity analysis results.

Condition	High Self-Image Cognition		Non-High Self-Image Cognition	
	Consistency	Coverage	Consistency	Coverage
Beauty Filter Use Habits	0.632195	0.62757	0.638821	0.6607
~Beauty Filter Use Habits	0.658199	0.636248	0.639905	0.644463
Beauty Filter Use Motivations	0.713999	0.723206	0.567639	0.599032
~Beauty Filter Use Motivations	0.604136	0.572857	0.737713	0.728808
Psychological Responses to Beauty Filter Use	0.731389	0.711679	0.583392	0.591439
~Psychological Responses to Beauty Filter Use	0.580122	0.572014	0.715602	0.735145
Beauty Filter Use Preferences	0.615195	0.616024	0.650275	0.678416
~Beauty Filter Use Preferences	0.67885	0.650726	0.631954	0.631138

Note. “~” denotes negation (absence of the condition).

**Table 11:** Configurational analysis results.

	High Self-Image Cognition		Non-High Self-Image Cognition		
	Configuration 1	Configuration 2	Configuration 1	Configuration 2	Configuration 3
Beauty Filter Use Habits		⊗	●		●
Beauty Filter Use Motivations	●		⊗	⊗	⊗
Psychological Responses to Beauty Filter Use	●	●	⊗	⊗	
Beauty Filter Use Preferences	⊗	●		●	●
Consistency	0.870		0.863		
Coverage	0.483		0.546		
Unique coverage	0.101	0.069	0.079	0.122	0.057
Solution consistency	0.886	0.903	0.905	0.897	0.891
Solution coverage	0.414	0.382	0.366	0.410	0.345

Note. ● indicates core presence; ● indicates peripheral presence; ⊗ indicates core absence; ⊗ indicates peripheral absence; Blank spaces indicate that the condition does not constitute a key condition.

## Data Availability

The original contributions presented in this study are included in the article. Further inquiries can be directed to the corresponding author.

## Supplementary Materials

The following supporting information can be downloaded at https://www.mdpi.com/article/10.3390/bs16071082/s1. Table S1: Complete Qualitative Coding Framework: Core Categories, Categories, Concepts, and Representative Raw Statements.
