# Supplementary material for: “Mirror, Mirror, Am I Beautiful?” Mechanisms of Self-Image Cognition and Behavioral Responses Among Chinese Youth in the Context of Digital Beauty Filter Use: A Mixed-Methods Study Using Grounded Theory and fsQCA
Source: Behav Sci (Basel). 2026 Jul 1;16(7):1082. doi: 10.3390/bs16071082 (PMC13405751; doi:10.3390/bs16071082)
Supplement: Supplementary file 1 [file behavsci-16-01082-s001.zip › behavsci-4288618-supplementary.pdf]

Table S1. Complete Qualitative Coding Framework: Core Categories, Categories, Concepts, and Representative Raw Statements

Core category	Category	Category description	Code	Concept	Representative raw statement
Beauty Filter Use Habits	A1 Use frequency	The distribution and regularity of how often beauty filters are used.	a1	High-frequency use	"I do it every time. With the original camera, I usually prefer a more retro tone, the kind with stronger contrast. Even if I do not retouch my face, I still adjust the tone to match the feel I like." (M02)
Beauty Filter Use Habits	A1 Use frequency	The distribution and regularity of how often beauty filters are used.	a2	Moderate-frequency use	"Unedited photos are usually landscapes, or some cute shots that are not of people. If it is a person, I usually do a bit of retouching." (F08)
Beauty Filter Use Habits	A1 Use frequency	The distribution and regularity of how often beauty filters are used.	a3	Low-frequency use	"Not very often, unless the school requires us to post group photos of students. I might post when I travel, but I still would not post personal photos on the platform." (M12)
Beauty Filter Use Habits	A2 Use contexts	The set of specific contexts in which beauty filters are used, including platform type, content type, and social circles.	a4	Platform context	"Definitely, because I think social media itself carries this desire to share." (F07)
Beauty Filter Use Habits	A2 Use contexts	The set of specific contexts in which beauty filters are used, including platform type, content type, and social circles.	a5	Content context	"If it is a person, I will do a bit of retouching. Even if it is not me and it is my friend posting it, I will still beautify it a little. I think it is a form of respect, respect for them." (F08)
Beauty Filter Use Habits	A2 Use contexts	The set of specific contexts in which beauty filters are used, including platform type, content type, and social circles.	a6	Social context	"What moves me most is social relationships, because people are not isolated individuals; it is about showing the good in oneself. For me, photos that show close relationships, such as taking a group photo together, and then beautifying it a bit, are more moving." (F12)
Beauty Filter Use Habits	A3 Use intensity	The degree to which beauty filters are used to modify one's appearance, ranging from light to moderate to heavy modification.	a7	Light retouching	"I do not do that kind of excessive fine editing; I just retouch it slightly." (M03)
Beauty Filter Use Habits	A3 Use intensity	The degree to which beauty filters are used to modify one's appearance, ranging from light to moderate to heavy modification.	a8	Moderate retouching	"For girls like us, the first thing I do is definitely make the head smaller and adjust the proportions. Then I start making targeted edits. I do not use ready-made templates from apps like Xingtu; instead, I manually adjust the areas I want to improve, such as the top of the hair, the shoulders, and posture. For girls, photo editing is a very complicated process. After editing, I will also think about whether to add a filter with more atmosphere." (F03)
Beauty Filter Use Habits	A3 Use intensity	The degree to which beauty filters are used to modify one's appearance, ranging from light to moderate to heavy modification.	a9	Excessive retouching	"When I first started editing photos, there was a period when I became quite obsessed with it. I would make my face very small." (M05)
Beauty Filter Use Habits	A4 Dependence on beauty filters	The level of reliance and stickiness in using beauty filters when posting appearance-related content, including high, moderate, and low dependence.	a10	High dependence	"It is not about following the crowd. I just feel that I must retouch it." (F04)
Beauty Filter Use Habits	A4 Dependence on beauty filters	The level of reliance and stickiness in using beauty filters when posting appearance-related content, including high, moderate, and low dependence.	a11	Moderate dependence	"I used to often take photos directly with a beauty camera. Later, to make them look more real, I chose to use the original camera and then edit the photo afterward. The photos come out with a different feeling. Beauty cameras make them look too overdone. If you take the photo with the original camera first and then retouch it a little, it feels more real and more consistent with your own features." (M06)
Beauty Filter Use Habits	A4 Dependence on beauty filters	The level of reliance and stickiness in using beauty filters when posting appearance-related content, including high, moderate, and low dependence.	a12	Low dependence	"During the period before the college entrance examination, I did not use beauty filters at all, because I felt they were too fake. They could not record my real life for myself, and I did not need to seek external evaluation. So I used the original camera completely." (F09)
Beauty Filter Use Preferences	A5 Feature preferences	Prioritized choices among feature types when using beauty filters, including basic retouching, advanced reshaping, and AI-generated functions.	a13	Basic features	"I think skin smoothing, skin smoothing, whitening, and face slimming." (M07)
Beauty Filter Use Preferences	A5 Feature preferences	Prioritized choices among feature types when using beauty filters, including basic retouching, advanced reshaping, and AI-generated functions.	a14	Advanced features	"Face editing in Xingtu? Xingtu is an app, and it has specific reshaping functions. It includes facial contours, such as the nose, eye size, facial outline, makeup, body shape, and filters. I adjust a bit of all of them." (F10)
Beauty Filter Use Preferences	A5 Feature preferences	Prioritized choices among feature types when using beauty filters, including basic retouching, advanced reshaping, and AI-generated functions.	a15	AI features	"In Douyin, in the AI image function, it will generate some very good-looking portrait photos for you!" (M08)
Beauty Filter Use Preferences	A6 Style preferences	Preferred directions and emphases among filter styles when enhancing appearance, such as natural, atmospheric, and refined styles.	a16	Natural style	"I feel that my editing skills have become better and better. Now my skills have reached a point where others cannot tell that I edited the photo, but I did edit it to restore a kind of original beauty." (F11)
Beauty Filter Use Preferences	A6 Style preferences	Preferred directions and emphases among filter styles when enhancing appearance, such as natural, atmospheric, and refined styles.	a17	Atmospheric style	"There is a popular word online now, 'atmosphere.' I will look at whether editing the photo this way gives it more atmosphere, making people feel something like a cinematic quality or another kind of feeling, and making the photo look better." (M09)
Beauty Filter Use Preferences	A6 Style preferences	Preferred directions and emphases among filter styles when enhancing appearance, such as natural, atmospheric, and refined styles.	a18	Refined style	"There was one time when I felt it was still relatively refined—or rather, a better kind of experience of filming a vlog and recording life—that really attracted me. I especially liked it." (F13)
Beauty Filter Use Preferences	A6 Style preferences	Preferred directions and emphases among filter styles when enhancing appearance, such as natural, atmospheric, and refined styles.	a19	Personalized style	"As for the refined socialite style you just mentioned, I actually do not really like that style. I prefer something with vitality. If I see photos on WeChat Moments or Douyin that feel full of life, energetic, and infectious, I tend to like that type more. A photo actually reflects a person's character. If the person is not lively and positive, they cannot really take a photo with that kind of vitality." (M10)
Beauty Filter Use Preferences	A7 Tool preferences	Preferences and priorities across tool types used for beautification, including dedicated beauty-camera apps, professional editing software, and AI-based beauty tools.	a20	Dedicated beauty-camera apps	"It is because of the beauty camera. Apps such as Douyin and beauty-camera apps sometimes come with built-in beautification when taking photos. For example, the system preset already makes the photo look good, so I may not use another editing app to refine it carefully." (F14)

Core category	Category	Category description	Code	Concept	Representative raw statement
Beauty Filter Use Preferences	A7 Tool preferences	Preferences and priorities across tool types used for beautification, including dedicated beauty-camera apps, professional editing software, and AI-based beauty tools.	a21	Professional photo-editing software	"I think the most important thing is still the filter. Once the face or body posture has been edited, I usually do not change it again; I only make further adjustments to the filter. I also use many apps, such as Filter Master, Dazz, and the recently released Copy. I switch among different apps to see which filter looks the best." (M11)
Beauty Filter Use Preferences	A7 Tool preferences	Preferences and priorities across tool types used for beautification, including dedicated beauty-camera apps, professional editing software, and AI-based beauty tools.	a22	AI tools	"What impressed me most was using Doubao AI. Recently, there was a popular trend of taking AI photos with game characters. I wanted to try it, and I found that the result was quite good. It broke the boundary between the two-dimensional and three-dimensional worlds." (M16)
Beauty Filter Use Motivations	A8 Self-presentation motivation	Purposive reasons for using beauty filters to achieve self-expression goals, such as idealized self-presentation and recording life moments.	a23	Idealized self-presentation	"On social platforms, after beautification, what is presented is an idealized version of oneself." (F02)
Beauty Filter Use Motivations	A8 Self-presentation motivation	Purposive reasons for using beauty filters to achieve self-expression goals, such as idealized self-presentation and recording life moments.	a24	Meeting presentation needs	"On social platforms you definitely want to show a better self, to build a so-called persona, so I will choose photos that have been retouched before posting." (F05)
Beauty Filter Use Motivations	A8 Self-presentation motivation	Purposive reasons for using beauty filters to achieve self-expression goals, such as idealized self-presentation and recording life moments.	a25	Ritualized life recording	"I share it partly to record my life, because I am someone who really likes posting to Moments. Every time I post, the photos have to look perfect." (F06)
Beauty Filter Use Motivations	A8 Self-presentation motivation	Purposive reasons for using beauty filters to achieve self-expression goals, such as idealized self-presentation and recording life moments.	a26	Presenting the authentic self	"I feel that the beautified version is closer to me, because I think photo editing is a technology for restoring beauty." (M13)
Beauty Filter Use Motivations	A9 Emotion-related motivation	Emotion-oriented motives for using beauty filters to meet emotional needs, such as gaining others' approval, alleviating appearance anxiety, or compensating for perceived image flaws.	a27	Gaining others' approval	"When I post on Moments and people like and comment that it looks good, I feel more confident." (F16)
Beauty Filter Use Motivations	A9 Emotion-related motivation	Emotion-oriented motives for using beauty filters to meet emotional needs, such as gaining others' approval, alleviating appearance anxiety, or compensating for perceived image flaws.	a28	Alleviating appearance anxiety	"She is very beautiful. She could even rely on her looks to eat out or work as a model. I am clearly not at that level. To be honest, there is some anxiety, but I feel better after adjusting myself." (F17)
Beauty Filter Use Motivations	A9 Emotion-related motivation	Emotion-oriented motives for using beauty filters to meet emotional needs, such as gaining others' approval, alleviating appearance anxiety, or compensating for perceived image flaws.	a29	Obtaining momentary pleasure	"Sometimes, right after I finish editing, I think it looks super good." (M14)
Beauty Filter Use Motivations	A9 Emotion-related motivation	Emotion-oriented motives for using beauty filters to meet emotional needs, such as gaining others' approval, alleviating appearance anxiety, or compensating for perceived image flaws.	a30	Positive self-motivation	"In terms of behavior, it may be a kind of encouragement for myself. For example, when I feel something about my skin condition or whether I am fat or thin, I may make a decision to improve myself appropriately in real life. As for filters, I do not really want to imitate others; it is more a kind of encouragement for my real self." (M18)
Beauty Filter Use Motivations	A9 Emotion-related motivation	Emotion-oriented motives for using beauty filters to meet emotional needs, such as gaining others' approval, alleviating appearance anxiety, or compensating for perceived image flaws.	a31	Stress release	"I feel that editing photos is a way to relieve stress. When I make the unattractive parts look better, I feel a sense of accomplishment." (M15)
Beauty Filter Use Motivations	A10 Social interaction motivation	Social-feedback-oriented motives for using beauty filters to increase social media likes, comment interactions, and other forms of social feedback.	a32	Need for everyday interaction	"The first point is that I expect others to interact with me and like my posts." (F19)
Beauty Filter Use Motivations	A10 Social interaction motivation	Social-feedback-oriented motives for using beauty filters to increase social media likes, comment interactions, and other forms of social feedback.	a33	Adaptation to group conformity	"My friends around me use it very frequently. They use it and then recommend it to me, so I also use it. That is how it is." (M16)
Beauty Filter Use Motivations	A10 Social interaction motivation	Social-feedback-oriented motives for using beauty filters to increase social media likes, comment interactions, and other forms of social feedback.	a34	Maintaining social recognition	"Later, after I post it, through people's likes and comments, I can infer which state is more likely to be recognized by others, or which state has failed. I will reflect on it and summarize it. So for me, it is more effective overall." (F20)
Beauty Filter Use Motivations	A10 Social interaction motivation	Social-feedback-oriented motives for using beauty filters to increase social media likes, comment interactions, and other forms of social feedback.	a35	Obtaining positive feedback	"I do care about it. I think it is also a kind of validation of my social ability. If I can obtain this positive feedback, it may have some influence on adjusting my social strategy, although it is not entirely the main influence." (M17)
Beauty Filter Use Motivations	A10 Social interaction motivation	Social-feedback-oriented motives for using beauty filters to increase social media likes, comment interactions, and other forms of social feedback.	a36	Aligning with platform aesthetic trends	"I think social media should be related to the aesthetic trends shaped by the media. The makeup and styling styles popular on media platforms each year or each quarter can play a guiding role. I think it is enough to have one's own standards." (F21)
Beauty Filter Use Motivations	A11 Instrumental rationality motivation	Instrumental reasons for using beauty filters to meet practical needs, such as correcting lens distortion and optimizing image color, lighting, and visual texture.	a37	Correcting lens distortion	"Because the lens still makes us look a little distorted, so you need to fix it." (M18)
Beauty Filter Use Motivations	A11 Instrumental rationality motivation	Instrumental reasons for using beauty filters to meet practical needs, such as correcting lens distortion and optimizing image color, lighting, and visual texture.	a38	Optimizing image texture	"Because I think with pure daylight, for example, if you take a photo indoors, it is either very dark or the tone is very monotonous, so I will do some simple editing and adjust the tone." (F22)

Core category	Category	Category description	Code	Concept	Representative raw statement
Psychological Responses to Beauty Filter Use	A12 Social comparison	Psychological states arising during beauty filters use, including comparisons with others' filtered images, benchmarking against one's ideal image, and efforts to avoid negative comparisons.	a39	Upward comparison	"That person is genuinely very good-looking, from bone structure to skin. Even if I edit my photo, it still does not look as good as their original, and that makes me feel a bit of appearance anxiety." (F15)
Psychological Responses to Beauty Filter Use	A12 Social comparison	Psychological states arising during beauty filters use, including comparisons with others' filtered images, benchmarking against one's ideal image, and efforts to avoid negative comparisons.	a40	Lateral comparison	"In everyday life, for example, my roommate has very fair skin. I often say, you are so white, and then I ask her to stretch out her arm. I am like an Oreo, the cream filling. I feel like the outer shell is real, and then you also think, why is my skin tone so dark?" (F20)
Psychological Responses to Beauty Filter Use	A12 Social comparison	Psychological states arising during beauty filters use, including comparisons with others' filtered images, benchmarking against one's ideal image, and efforts to avoid negative comparisons.	a41	Downward comparison	"Once, I saw that our vice class monitor used a beauty filter and looked especially unattractive. We laughed about it for a long time, and I thought I would never use that beauty filter again." (M19)
Psychological Responses to Beauty Filter Use	A13 Defensive psychology	Defensive tendencies formed after using beauty filters to manage potential risks, such as coping with negative evaluations or concealing perceived flaws.	a42	Blocking negative evaluations	"I would scold them in the comments section, and after that I would not really care. I just do not pay much attention to comments like that. They may create some thoughts in my mind, but they would not make me do anything in particular. I would not delete the comment, nor would I delete the Moments post." (F23)
Psychological Responses to Beauty Filter Use	A13 Defensive psychology	Defensive tendencies formed after using beauty filters to manage potential risks, such as coping with negative evaluations or concealing perceived flaws.	a43	Concealing flaws	"Because I have quite a lot of acne on my face, I will edit the acne a bit." (M20)
Psychological Responses to Beauty Filter Use	A13 Defensive psychology	Defensive tendencies formed after using beauty filters to manage potential risks, such as coping with negative evaluations or concealing perceived flaws.	a44	Image maintenance	"Since I post it, it must have reached a level I am satisfied with. For example, if I think my forehead is very large, I definitely will not allow myself to post a photo with such a big forehead. If I were to post one with a big forehead, I might as well not post it." (F24)
Psychological Responses to Beauty Filter Use	A14 Ambivalence	Ambivalent psychological states at the cognitive and behavioral levels, shaped by dependence on filter effects alongside vigilance toward possible cognitive distortion.	a45	Desire for recognition vs. fear of falseness	"After editing a photo, I usually clearly recognize that the picture is fake. Every time I post, I feel conflicted. On the one hand, I hope to gain others' recognition. On the other hand, I feel uneasy about whether others will think the adjustments are a bit excessive. Under this psychological effect, I rarely post." (M21)
Psychological Responses to Beauty Filter Use	A14 Ambivalence	Ambivalent psychological states at the cognitive and behavioral levels, shaped by dependence on filter effects alongside vigilance toward possible cognitive distortion.	a46	Technological dependence vs. vigilance toward alienation	"I think I rely too much on AI. Almost every photo I post has been edited. I have thought about posting unedited photos, but maybe because all the photos I posted before were edited, I feel a bit burdened, a kind of psychological burden. I feel that if this photo is too different from the ones I posted before, I do not really dare to post it." (F01)
Psychological Responses to Beauty Filter Use	A14 Ambivalence	Ambivalent psychological states at the cognitive and behavioral levels, shaped by dependence on filter effects alongside vigilance toward possible cognitive distortion.	a47	Pursuit of perfection vs. acceptance of imperfection	"I cannot accept myself without beauty filters. I feel that I do not look like myself at all. Maybe I have too strong a filter for myself. I feel that the original photo simply does not look like me; it is not me." (M01)
Psychological Responses to Beauty Filter Use	A14 Ambivalence	Ambivalent psychological states at the cognitive and behavioral levels, shaped by dependence on filter effects alongside vigilance toward possible cognitive distortion.	a48	Self-display vs. privacy protection	"I am not very willing to let others know the real me... I have a sense of confidentiality... I do not really want unrelated people to know too much about my life." (M04)

Note. Category and concept labels are translated in alignment with the English manuscript. Raw interview excerpts are translated from participants' original statements, with participant IDs retained.
